# Plant caspase-like proteins: from function identification to application in winter rapeseed genetic breeding

**DOI:** 10.3389/fpls.2026.1858423

**Published:** 2026-06-03

**Authors:** Tingting Fan, Abbas Muhammad Fahim, Li Ma, Lijun Liu, Yuanyuan Pu, Wangtian Wang, Wancang Sun, Gang Yang, Junyan Wu

**Affiliations:** 1 College of Agronomy, State Key Laboratory of Arid Land Crop Science, Gansu Agricultural University, Lanzhou, China; 2 Gansu Agricultural University, Seed Industry Research Institute of Gansu Provincial University, Lanzhou, China; 3 College of Life Science and Technology, Gansu Agricultural University, Lanzhou, China

**Keywords:** caspase-like proteins, genetic breeding, metacaspase, programmed cell death, vacuolar processing enzymes, winter rapeseed

## Abstract

Plant programmed cell death (PCD) shares striking similarities with animal apoptosis in both morphological and biochemical characteristics, yet plant genomes lack genuine orthologs of animal caspases. Instead, plants have evolved a category of caspase-like proteins that are functionally analogous but lack sequence homology with animal caspases. This review systematically summarizes recent advances in the research of major plant caspase-like proteins, including metacaspases, vacuolar processing enzymes (VPEs), saspases, phytaspases, the proteasomal β subunit PBA1, and cathepsin B. These proteins play pivotal regulatory roles in PCD triggered during plant development, senescence, biotic and abiotic stresses, and exhibit distinctive substrate specificities, activation mechanisms, and regulatory networks. Furthermore, focusing on winter rapeseed, this review discusses the application potential of caspase-like proteins in genetic breeding, such as enhancing stress resistance by modulating their activities, optimizing yield-related traits, and improving biotechnological breeding platforms including microspore embryogenesis. Despite challenges including functional redundancy, spatiotemporal regulation, and species-specific divergence, caspase-like proteins serve as core nodes in the PCD regulatory network and provide valuable targets for crop improvement. This review offers a systematic reference for further understanding the molecular mechanisms of plant PCD and its application in crop breeding.

## 1 Introduction

Research into plant programmed cell death (PCD) has spanned over three decades, revealing striking similarities with animal apoptosis. These conserved hallmarks include cell shrinkage, nuclear condensation, DNA laddering, mitochondrial membrane permeabilization leading to cytochrome c release, reactive oxygen species (ROS) bursts, and elevated caspase-like enzymatic activity (Dickman et al., 2017). The presence of structures resembling apoptotic bodies further underscores these parallels. Despite the divergence in the core executioners-as plants lack true orthologs of animal caspases-these shared features suggest the existence of fundamental regulatory principles underlying PCD across kingdoms. The well-established mechanistic framework of animal apoptosis has thus served as a valuable guide for exploring PCD in plants.

In animals, caspases (cysteinyl aspartate-specific proteases) are the quintessential executioners of apoptosis, irreplaceable in initiating, amplifying, and executing cell death through the specific cleavage of substrates after aspartic acid residues. The surprising absence of caspase orthologs in plant genomes indicates that plants have evolved a distinct mechanistic solution (Huh, 2022; Beers et al., 2000). This role is fulfilled by a suite of functionally analogous proteases, collectively termed caspase-like proteins, which exhibit similar specificity and drive PCD despite lacking sequence homology to their animal counterparts. This review systematically summarizes recent advances in our understanding of these plant caspase-like proteins, focusing on their biochemical characteristics, regulatory mechanisms, and diverse roles in PCD (Wleklik and Borek, 2023; Tran et al., 2014; Štrancar et al., 2022). Notably, winter rapeseed as an important oilseed crop, frequently suffers from yield limitations caused by PCD-related abiotic and biotic stresses. Therefore, elucidating the regulatory roles of caspase-like proteins also provides valuable molecular targets for genetic breeding to enhance stress resistance and optimize agronomic traits (Kourani et al., 2025).

## 2 Caspases are key regulators and executors of PCD in animals

Caspases are a family of cysteine-dependent aspartate-specific proteases (Sahoo et al., 2023). Their role as regulators of cell death was established over three decades ago with the discovery of CED-3 (Conradt et al., 2016), the protein that executes developmental cell death in *Caenorhabditis elegans*. Mammalian homologs include at least 18 members (varying by species) and are functionally divided into three subgroups (Julien and Wells, 2017):

Inflammation and pyroptosis-related: caspase-1, -4, -5, -11, -12, mediating inflammatory responses and pyroptosis. Apoptosis initiators: caspase-2, -8, -9, -10, which auto-activate in response to upstream signals and subsequently activate downstream effector molecules. Apoptosis executioners: caspase-3, -6, -7, which cannot auto-activate and are activated solely by upstream caspases to cleave key substrates, inducing the morphological and biochemical changes characteristic of apoptosis (Grant and Boucher, 2025; Martinvalet, 2019).

## 3 Plants lack caspase orthologs

Given the crucial role of caspases in animal cell death, botanists systematically searched for potential orthologs during their investigation of plant programmed cell death (PCD). Surprisingly, no true caspase orthologs were identified in plants (Sueldo and van der Hoorn, 2017; Salvesen et al., 2016; Bonneau et al., 2008; Huh, 2022). Subsequently, Uren et al. used PSI-BLAST sequence alignment to discover metacaspases in *Arabidopsis thaliana* and other plant species, which share structural similarities with caspases (Uren et al., 2000). However, significant debate persists among botanists regarding whether metacaspases are functional analogues of caspases.

Some researchers posit that metacaspases are the functional equivalents of caspases in plants, potentially representing ancestral caspase genes (Minina et al., 2017). This view is supported by evidence of metacaspase involvement in various plant PCD processes (Watanabe and Lam, 2005; Suarez et al., 2004; Bozhkov et al., 2005) and their ability, like caspases, to cleave the phylogenetically conserved substrate Tudor staphylococcal nuclease (TSN) (Gutierrez-Beltran et al., 2016).

Conversely, other scholars argue that metacaspases are not functional analogues. They reason that although metacaspases are cysteine-dependent hydrolases, they cannot cleave typical caspase substrates, and their proteolytic activity is resistant to caspase-specific inhibitors (Basak and Kundu, 2022). Despite this ongoing debate, it remains an established fact that no true caspase orthologs exist in plants.

## 4 Activation of caspase-like activity during plant PCD

In 1998, botanists detected a proteolytic activity in tobacco that cleaves the synthetic caspase-1 substrate Ac-YVAD-MCA and found it to be essential for bacteria-induced PCD (del Pozo and Lam, 1998). This study was the first to establish a link between caspase-like protein activity and plant cell death. Although no true caspase orthologs exist in plants, subsequent research has revealed elevated caspase-like activity during PCD in numerous plant species using synthetic caspase tetrapeptide fluorescent substrates. Furthermore, studies employing caspase inhibitors have demonstrated that this increased activity is necessary for plant PCD to occur (Van Durme and Nowack, 2016; Bonneau et al., 2008). Given the absence of genuine caspases, this detected enzymatic activity is termed “caspase-like activity.” Among these, caspase-1-like and caspase-3-like activities have been the most extensively studied, with the elevation of caspase-3-like activity often serving as a key indicator of PCD in plants.

In animal cells, caspase-3 typically exists as an inactive proenzyme and only becomes functional after being cleaved and activated by upstream caspases (Sychta et al., 2021). Although caspase-3-like activity is significantly elevated during various types of plant PCD, its activation mechanism remains poorly understood. For instance, during heat shock-induced PCD, heat stress triggers an increase in intracellular ROS levels, which activates MPK6 via Ca^2^^+^ signaling. This cascade promotes the expression and activation of the vacuolar processing enzyme (VPE), which mediates vacuole rupture and ultimately leads to elevated caspase-3-like activity (Li et al., 2012). Additionally, a more recent study found that overexpression of the NAC089 transcription factor can induce an increase in caspase-3-like activity (Yang et al., 2014). However, the specific protein responsible for this caspase-3-like activity in these pathways remains unidentified.

Given the discovery of caspase-like activity and its functional significance in plant PCD, botanists have long sought to identify the proteins responsible. Identifying these proteases is critical for advancing PCD research. After more than a decade of investigation, researchers have identified multiple plant proteins that exhibit caspase-like activity and play important roles in PCD (Sueldo and van der Hoorn, 2017; Salvesen et al., 2016; Coppola et al., 2024).

## 5 The function of metacaspase in plant PCD

### 5.1 Structural similarity and enzymatic divergence of metacaspases

**Figure 1:**
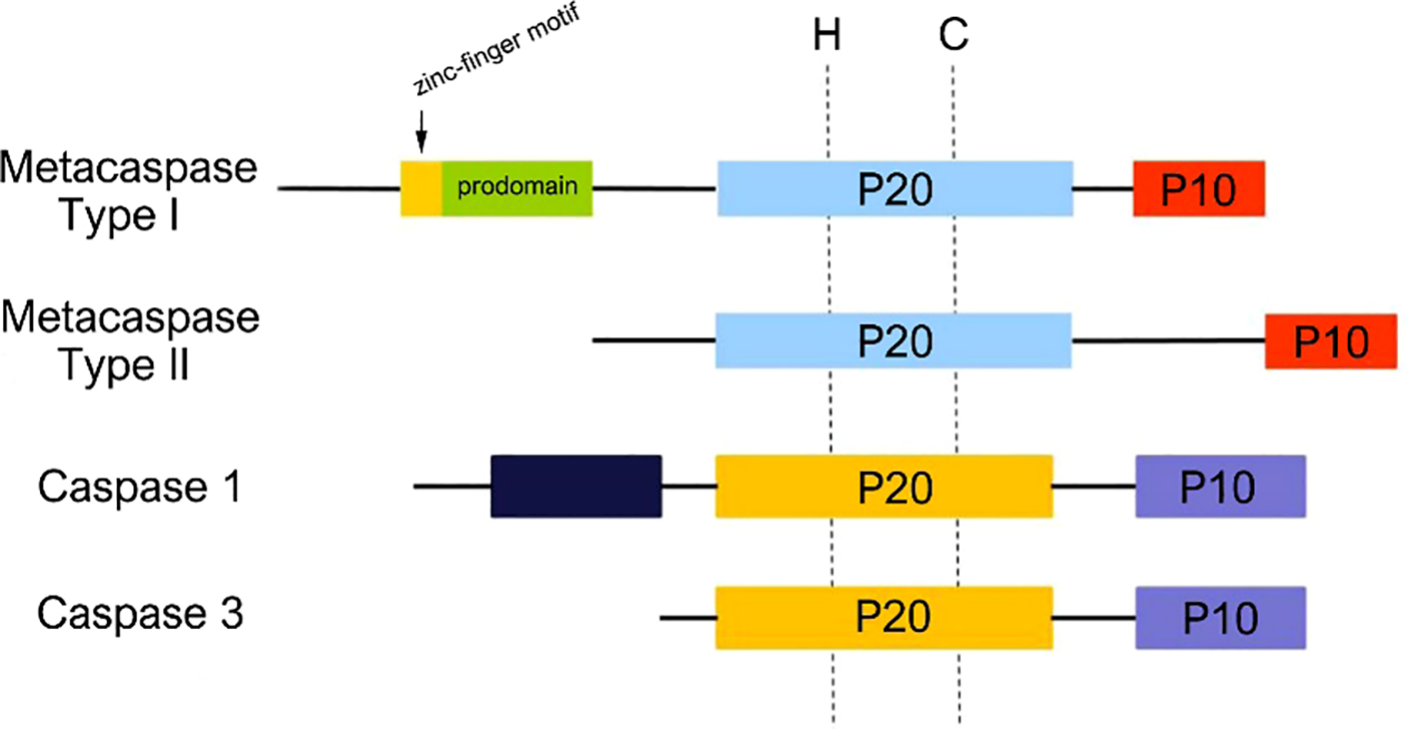
Diagram of metacaspases and caspases (Cai, 2013; Klemenčič and Funk, 2019; Minina et al., 2017). Two type of metacaspases in *Arabidopsis thaliana* and two types of caspases in animal cells are shown in the above **Figure 1**. Different color blocks represent different domains. Yellow and green blocks are metacaspases pro-domain, blue blocks are metacaspases large subunit P20 domain, red blocks are metacaspases small subunit P10 domain, dark block is caspases pro-domain, orange blocks are caspases P20 domain and purple blocks are caspases P10 domain. H is Histidine acids residue and C is Cysteine acids residue.

Although the functional equivalence of metacaspases to caspases remains debated, they are the only plant proteins with significant structural and sequential homology, as both belong to the C14 family of cysteine proteases (Huh, 2022). Metacaspases contain the characteristic P20 and P10 structural domains, with the P20 domain harboring the essential histidine-cysteine (His-Cys) catalytic dyad (**Figure 1**) (Klemenčič and Funk, 2019; Minina et al., 2017). Notably, metacaspases are found exclusively in species that lack true caspases, such as plants, fungi, and yeast (Huh, 2022).

The *Arabidopsis thaliana* genome encodes nine metacaspases (AtMC1-9), classified into Type I and Type II based on structural differences (**Figure 1**) (Tsiatsiani et al., 2011; Cai, 2013). Type I metacaspases contain an N-terminal prodomain with a proline-rich region and a zinc finger motif, while Type II lacks this domain. The catalytic core of both types consists of large (P20) and small (P10) subunits and the conserved His-Cys dyad. These features initially suggested that metacaspases might exhibit functional and enzymatic activities analogous to caspases.

Early support for this idea came from a study of a Norway spruce metacaspase, which was found to regulate VEIDase activity, a caspase-6-like function *(*Bozhkov et al., 2005). However, subsequent enzymatic studies revealed a different reality. Vercammen et al. demonstrated that recombinant AtMC4 and AtMC9 cleave the canonical caspase substrate DEVD with less than 5% efficiency, compared to 95% for human caspase-7. Instead, they exhibited high cleavage efficiency for substrates with arginine (R) at the P1 position (Vercammen et al., 2004). This finding was corroborated by studies on AtMC8, which showed strong activity for a GRR substrate but no activity for DEVD or VEID (He et al., 2008). Similarly, the Type I metacaspase AtMC1 was found to preferentially cleave after arginine or lysine residues (Watanabe and Lam, 2005). Collectively, these studies clearly demonstrate that metacaspases lack typical caspase-like proteolytic activity.

### 5.2 The pivotal role of metacaspases in plant PCD

Despite their divergent substrate specificity, metacaspases are indispensable regulators of plant programmed cell death (PCD). Their involvement was first established in Norway spruce embryogenesis, where suspensor PCD is arrested upon metacaspase dysfunction (Bozhkov et al., 2005). This discovery spurred extensive research linking metacaspases to diverse PCD contexts.

**Figure 2:**
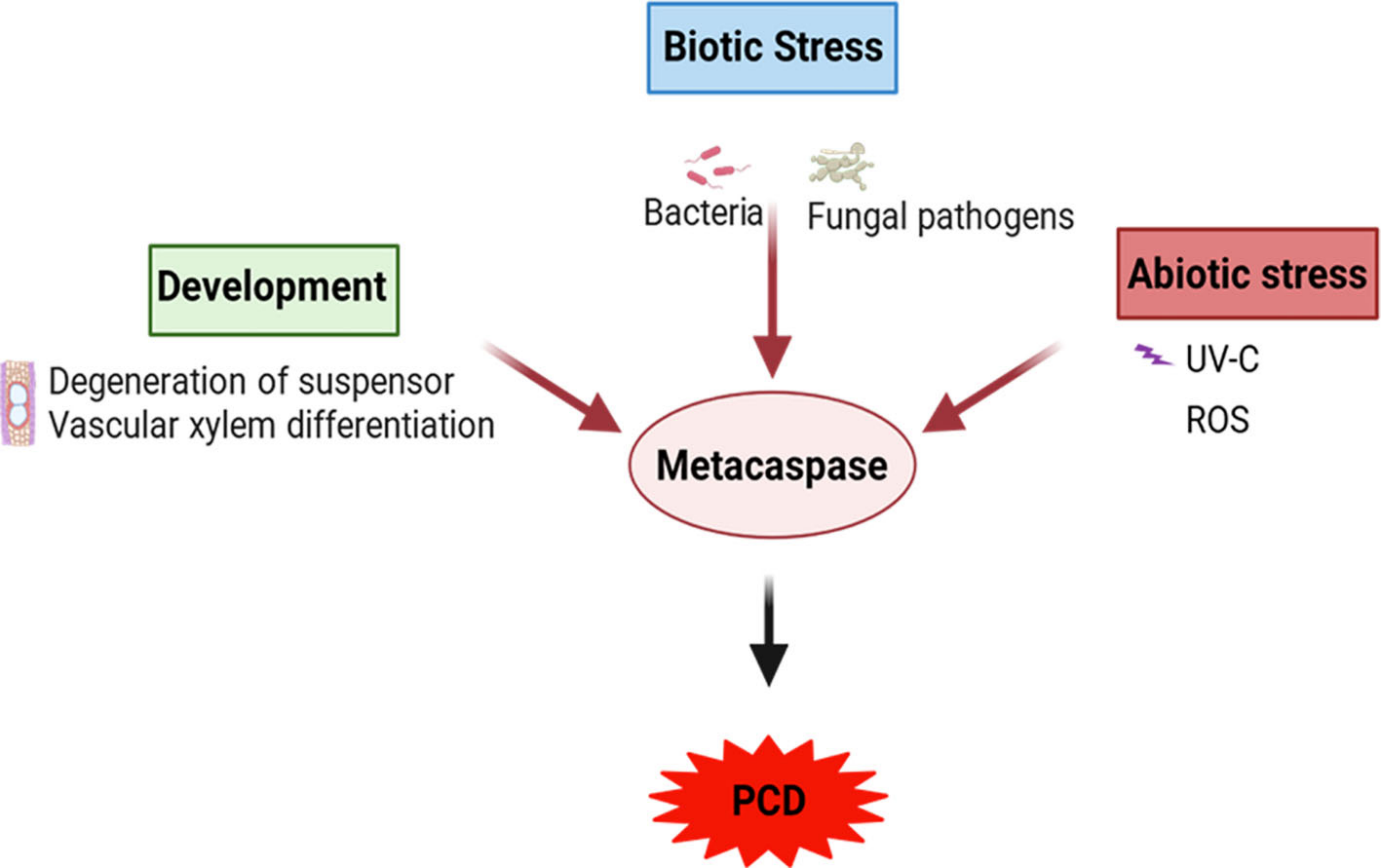
Summary of the induction, process and functions of metacaspase in plants. Metacaspase is induced by biotic stress (e.g., pathogen infection), abiotic stress (e.g., UV−C, H_2_O_2_, oxidative stress), and developmental signals (e.g., vascular xylem differentiation, embryogenesis involving suspensor degeneration). Upon activation, metacaspase regulates programmed cell death (PCD) in these contexts.

- **Developmental PCD:** In Norway spruce, mcII-Pa activates autophagy during suspensor PCD (Garcia et al., 2022), a role mirrored in *Arabidopsis*, where AtMC9 regulates autophagy to suppress or execute cell death during vascular xylem differentiation (**Figure 2**) (Escamez et al., 2016; Bollhöner et al., 2013; Iakimova et al., 2024).
- **Abiotic stress-induced PCD:** AtMC8 expression is upregulated by UV-C and H_2_O_2_ stress. Accordingly, *AtMC8* knockout reduces PCD, while its overexpression enhances sensitivity to these stressors (**Figure 2**) (He et al., 2008). Similarly, AtMC4 acts as a positive regulator of PCD induced by oxidative stressors like the fungal toxin fumonisin B1, methyl viologen, and acifluorfen (Watanabe and Lam, 2011).
- **Biotic stress-induced PCD:** Metacaspases also modulate pathogen response (**Figure 2**). Knockout of *AtMC4* reduces PCD triggered by *Pseudomonas syringae* (Watanabe and Lam, 2011). Intriguingly, different metacaspases can have opposing effects; in the *lsd1* mutant, AtMC1 promotes PCD induced by a salicylic acid analog, while AtMC2 suppresses it (Coll et al., 2010). Further research confirmed that AtMC1 positively regulates hypersensitive response (HR)-induced cell death, operating in parallel with autophagy pathways (Coll et al., 2014).

### 5.3 Activation and regulation of metacaspases

The activation of metacaspases is a critical control point for their function. Like caspases, they are synthesized as inactive proenzymes that require proteolytic processing. Metacaspases can undergo autoactivation, as demonstrated by the spontaneous processing of recombinant AtMC9 into its P20 and P10 fragments (Liu et al., 2025). This autoactivation is often dependent on Ca^2+^ binding (Garcia et al., 2022; Fernández-Fernández et al., 2024). Metacaspase activity is susceptible to inhibition by zinc ions (Helmersson et al., 2008), specific cysteine modifications like S-nitrosylation (Lema Asqui et al., 2018), and protease inhibitors such as AtSerpin1, which inhibits both AtMC9 and AtMC1 (Fernández-Fernández et al., 2023).

### 5.4 Substrate spectrum of metacaspases

Identifying metacaspase substrates is key to understanding their mechanistic roles. Their substrates can be broadly categorized as follows:

- **PCD-related substrates:**
- **AtSerpin1:** The first identified substrate; AtMC9 cleaves this serine protease inhibitor, which then forms an inhibitory complex with the metacaspase (Lema Asqui et al., 2018).
- **TSN:** The Tudor staphylococcal nuclease, a known caspase-3 substrate in animals, is cleaved by a Norway spruce metacaspase (Sundström et al., 2009).
- **GRI (GRIM REAPER):** Cleavage by AtMC9 releases an 11-amino acid peptide that activates the kinase PRK5, inducing ROS-triggered cell death (Wrzaczek et al., 2015). This was the first evidence of metacaspases processing extracellular signals.
- **PROPEP1:** AtMC4 cleaves this precursor to produce the immune signal peptide Pep1 during tissue damage and pathogen attack (Hander et al., 2019; Shen et al., 2019; Shalini et al., 2015).

**Non-PCD and metabolic substrates:**

- **PEPCK1:** AtMC9 cleaves phosphoenolpyruvate carboxykinase 1 to enhance its activity, positively regulating gluconeogenesis. This indicates metacaspase functions beyond cell death (Rojas et al., 2021).
- **Other potential targets:** Proteomic studies suggest a wider range of substrates, including 14-3–3 proteins, aspartic proteases, and other hydrolases, though direct validation is often needed (Tsiatsiani et al., 2013; Bollhöner et al., 2018).

### 5.5 Bridging metacaspase to other caspase-like proteins and breeding applications

Collectively, metacaspases are central regulators of plant PCD with diverse activation mechanisms and substrates. However, they are not the only caspase-like proteins in plants. As detailed below, VPEs, saspases, phytaspases, PBA1, and cathepsin B each contribute to PCD with distinct specificities. Understanding this functional network is essential for crop improvement. In winter rapeseed, where PCD affects stress tolerance, yield, and microspore embryogenesis, manipulating these proteases offers a promising strategy, as discussed in Sections 10–11.

## 6 The function of VPEs in plant PCD

### 6.1 The discovery and characteristics of VPEs

In 1987, Japanese botanists identified a protease in pumpkin seeds responsible for maturing storage proteins within seed vacuoles (Hara-Nishimura and Nishimura, 1987). This enzyme, later purified from castor bean seeds in 1991, was officially named Vacuolar Processing Enzyme (VPE) (Hara-Nishimura et al., 1991). VPE is the plant homolog of legumain and a member of the C13 family of cysteine proteases (Hara-Nishimura et al., 1993), representing one of the earliest and most extensively studied plant cysteine proteases. The *Arabidopsis thaliana* genome encodes four VPE isoforms-αVPE, βVPE, γVPE, and δVPE-which are categorized based on expression patterns into vegetative-type (αVPE and γVPE) and seed-type (βVPE and δVPE) (Hara-Nishimura and Hatsugai, 2011).

**Figure 3:**
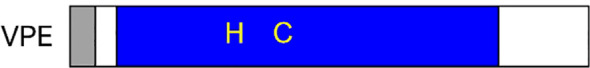
Diagram of VPE in plant. VPE have a signal peptide and a prodomain at the N-termini, and have a prodomain at the C-termini. Gray block is signal peptide, white block is prodomain and blue block is mature enzymes. H and C is Histidine and Cysteine acids residue, respectively.

VPE is synthesized as a preproenzyme precursor containing an N-terminal signal peptide and N- and C-terminal prodomains (**Figure 3**) Following translation, the precursor is directed to the endoplasmic reticulum, where the signal peptide is cleaved, forming an inactive proenzyme. This proenzyme is then transported through the secretory pathway to the cell wall or vacuoles (Vorster et al., 2019). Within the acidic environment of the vacuole, the VPE proenzyme undergoes autoactivation, shedding its prodomains to become a mature, active protease (Hiraiwa et al., 1999; Müntz and Shutov, 2002). The mature enzyme primarily cleaves peptide bonds after asparagine (Asn) and, to a lesser extent, aspartic acid (Asp) residues (Hiraiwa et al., 1999; Becker et al., 1995).

Notably, VPE exhibits caspase-1-like activity, making it the first identified plant cysteine protease with such a function (Hatsugai et al., 2004). This role was confirmed through multiple lines of evidence. Hatsugai et al. demonstrated that the caspase-1 inhibitor biotin-YVAD-FMK failed to label proteins in tobacco leaves with suppressed VPE expression and in protein extracts from *Arabidopsis* δVPE knockout seeds. Furthermore, recombinant VPE activity was abolished by incubation with Ac-YVAD-FMK (Nakaune et al., 2005). In a separate study, Qiang et al. observed reduced caspase-1-like activity in the roots of *Arabidopsis* vpe mutants during colonization by the symbiotic fungus Piriformospora indica (Qiang et al., 2012). Together, these findings robustly demonstrate that VPE functions as a principal caspase-1-like protease in plants.

### 6.2 The function of VPEs

**Figure 4:**
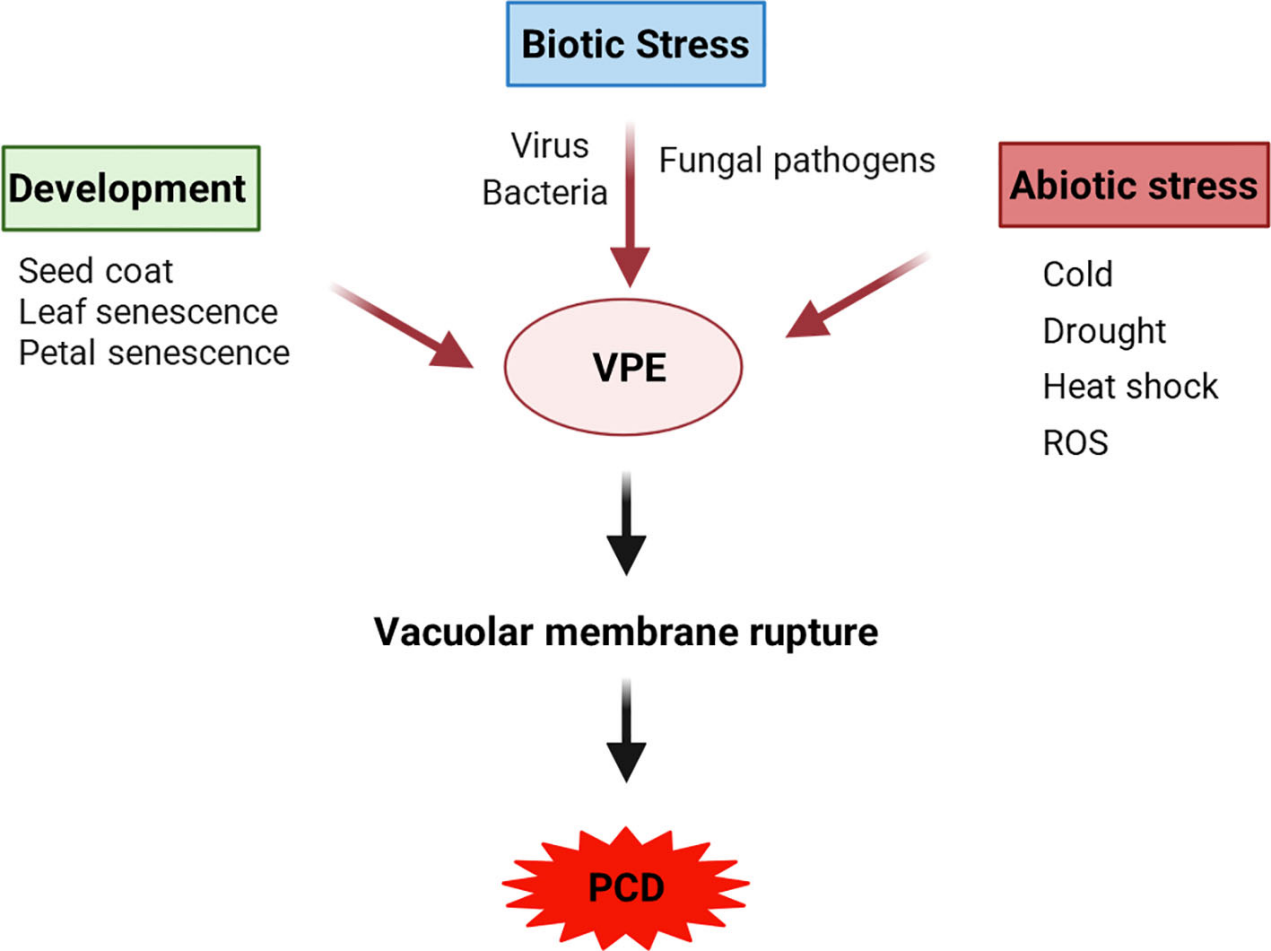
Summary of the induction, process and functions of VPE in plants. Vacuolar processing enzymes (VPEs) are induced by biotic stress (e.g., TMV infection, fungal toxin fumonisin B1, bacterial elicitors), abiotic stress (e.g., drought, salt, heat shock), and developmental signals (e.g., seed coat formation, leaf and petal senescence, loss of apical dominance). Upon activation in acidic vacuoles, VPEs trigger vacuolar membrane rupture – a hallmark of plant PCD – and execute programmed cell death in these contexts.

Vacuolar processing enzymes (VPEs) are crucial regulators of plant growth, development, and responses to biotic and abiotic stresses (**Figure 4**). During seed germination, VPEs mobilize storage proteins through direct proteolysis or by activating other peptidases (Hara-Nishimura and Hatsugai, 2011; Vorster et al., 2019; Cheng et al., 2020). A well-established function of VPEs is their role as key mediators of plant programmed cell death (PCD) (Wleklik and Borek, 2023; Teper-Bamnolker et al., 2021). A defining feature of plant PCD is the collapse of the vacuole. Due to the constraints of the cell wall, plant cells cannot be phagocytosed like animal cells. Instead, VPEs trigger the rupture of the vacuolar membrane, releasing hydrolases into the cytoplasm that initiate a proteolytic cascade, leading to rapid cellular dismantling (Hara-Nishimura et al., 2005).

#### 6.2.1 VPEs in developmental PCD

VPEs orchestrate PCD in various developmental contexts. In angiosperms, δVPE is expressed in the maternal tissues of the seed coat, where it induces PCD to reduce cell layer thickness and facilitate proper seed coat formation (Nakaune et al., 2005). Similarly, barley VPE4 mediates the degradation of maternal nucellus and seed coat tissues, directly influencing final seed size (Julián et al., 2013; Tran et al., 2014; Radchuk et al., 2018). VPEs also regulate developmental processes like apical dominance in potatoes, where low-temperature-induced PCD in the shoot apical meristem leads to its loss. Inhibiting VPE with Ac-YVAD-CHO accelerates tuber development while maintaining apical dominance (Teper-Bamnolker et al., 2017). Furthermore, VPEs contribute to leaf and petal senescence (Wleklik and Borek, 2023), and in rice, the expression of OsVPE2 and OsVPE3 is induced by H_2_O_2_ in a manner suppressible by the human anti-apoptotic protein BCL2, confirming their conserved role in PCD regulation (Deng et al., 2011).

#### 6.2.2 VPEs in biotic stress-induced PCD

VPEs are essential executors of PCD during plant immune responses. Silencing VPE in tobacco significantly reduces hypersensitive cell death induced by Tobacco Mosaic Virus (TMV) and prevents the characteristic vacuolar membrane rupture. VPE is also critical for effector-triggered immunity (ETI); for instance, silencing tobacco VPE inhibits PCD induced by the bacterial effector harpins and disrupts harpintriggered stomatal closure (Zhang et al., 2010). Similarly, VPE mediates PCD induced by the fungal toxin fumonisin B1. In *Arabidopsis*, knockout of VPE, particularly γVPE, alleviates fumonisin B1-induced PCD and vacuolar rupture, with the quadruple vpe mutant showing the strongest resistance (Kuroyanagi et al., 2005). Finally, VPE is required for the symbiotic relationship with the root-colonizing fungus *Piriformospora indica*, as knocking out VPE blocks the host cell PCD necessary for successful fungal colonization (Qiang et al., 2012).

#### 6.2.3 VPEs in abiotic stress responses

VPEs also function in abiotic stress adaptation. Under drought stress, various cysteine proteases, including VPE, are induced (Cilliers et al., 2018; Martinez and Diaz, 2008; Khanna-Chopra et al., 1999). In soybean nodules, VPE contributes to the drought stress response, and *Arabidopsis* vpe mutants under drought show reduced activity of C1A cysteine proteases, increased biomass, and higher protein content, suggesting VPE may activate other proteases in this pathway (Cilliers et al., 2018). The role of VPE in drought tolerance is highlighted by the fact that mutations in γVPE, which is highly expressed in guard cells, significantly enhance drought tolerance in *Arabidopsis* (Wleklik and Borek, 2023). In rice, inhibiting OsVPE3 enhances salt tolerance by reducing PCD-associated vacuolar rupture and modifying leaf anatomy (Lu et al., 2016).

## 7 The function of saspase and phytaspase in plant PCD

### 7.1 Saspases and phytaspases: serine proteases with caspase-like activity

The search for proteases mediating caspase-like activity in plants led to the discovery of two distinct groups within the serine protease family: saspases and phytaspases.

#### 7.1.1 Saspases

In 2004, Coffeen and Wolpert identified two novel serine proteases in oats with aspartic acid (Asp) specificity, naming them “saspases” (serine aspartic acid-specific proteases, SAS-1 and SAS-2) (Coffeen and Wolpert, 2004). These ~84 kDa enzymes are specifically inhibited by Ac-YVAD-CMK and cleave caspase-1, -6, and -8 substrates (YVAD, VKMD, IETD), but not the caspase-3 substrate DEVD (Coffeen and Wolpert, 2004). Saspases are active at an optimal pH of 5.5-6.5 and are released into the extracellular space during victorin and heat shock-induced PCD (Coffeen and Wolpert, 2004). Finally, recent studies suggest that the function of saspase may involve a broader signaling cascade network. In addition to directly cleaving specific substrates, it may also act synergistically with other serine proteases such as phytaspase to amplify programmed cell death (PCD) signals, thereby triggering rapid cellular disassembly (Wleklik and Borek, 2023).

#### 7.1.2 Phytaspases

Subsequently, a similar Asp-specific serine protease, named phytaspase, was discovered in tobacco and rice (Chichkova et al., 2010). Phytaspase preferentially binds the caspase-6 substrate VEID and is a key regulator of PCD. Silencing phytaspase reduces TMV-triggered hypersensitive cell death, while its overexpression exacerbates it (Cai et al., 2014). It also modulates oxidative stress-induced PCD, with overexpression increasing sensitivity to methyl viologen and downregulation conferring resistance (Chichkova et al., 2010). A significant functional parallel to animal caspases was demonstrated by its ability to cleave the TATD site in.

the *Agrobacterium tumefaciens* VirD2 protein, a known substrate of human caspase-3 (Chichkova et al., 2010, Chichkova et al., 2004). This caspase-like activity has been consistently confirmed in various experimental systems (Galiullina et al., 2015; Chichkova et al., 2014). In addition, phytaspase exhibits broader functions in plant responses to abiotic stresses (such as high salinity, drought, and low temperature) as well as biotic stresses (including pathogen infection). It is regarded as a critical node linking stress signaling to the execution machinery of cell death, and plays a central role particularly in the hypersensitive response (HR) induced by pathogen infection, such as viral attack (Kacprzyk et al., 2024).

### 7.2 Evolutionary context and broader functions of plant subtilases

Phylogenetically, both saspases and phytaspases belong to the subtilase family (S8 family in the MEROPS database) of serine proteases, which feature a catalytic triad of aspartate, histidine, and serine A typical plant subtilase consists of a secretory signal peptide, a prodomain, and a peptidase S8 domain (Vartapetian et al., 2011; Rawlings et al., 2008). The *Arabidopsis thaliana* genome encodes 56 subtilases, divided into six subgroups (Serrano et al., 2016). Phytaspases from tobacco and rice, along with the rice subtilase most similar to oat saspase, cluster within *Arabidopsis* subgroup I (Vartapetian et al., 2011). To date, only one *Arabidopsis* gene, *AtPhyt* (*Arabidopsis thaliana phytaspase*), has been confirmed to encode a phytaspase (Chichkova et al., 2018).

Functional studies of AtPhyt revealed that its substrate specificity is environmentally regulated: it is strictly Asp-specific under acidic conditions but hydrolyzes substrates after His and Phe residues at neutral pH (Chichkova et al., 2018). AtPhyt localizes to the apoplast and may be internalized into cells via clathrin-mediated endocytosis (Chichkova et al., 2018; Trusova et al., 2019). The functional repertoire of phytaspases extends beyond PCD. In tomatoes, 12 phytaspase (*SlPhyt*) genes have been identified, with several exhibiting caspase-like activity and the ability to induce cell death upon ectopic expression (Reichardt et al., 2018). Crucially, some tomato phytaspases process peptide hormone precursors; they cleave prosystemin to generate systemin and process the precursor of phytosulfokine (PSK) to promote drought-induced flower abscission (Beloshistov et al., 2018; Reichardt et al., 2020). A recent interactome study also identified endoplasmic reticulum proteins like calreticulin-3 as potential phytaspase interaction partners, suggesting a role in senescence-related processes (Teplova et al., 2021).

## 8 The function of proteasome β1 subunit PBA1 in plant PCD

For a long time, the protease responsible for caspase-3-like activity in plants has perplexed plant scientists. In 2009, the first protein with caspase-3-like activity identified in plants was the 20S proteasome β1 subunit, PBA1. The use of proteasome-specific inhibitors, β-lactone and aPnLd, significantly reduced caspase-3-like activity in total leaf protein extracts (Üstün et al., 2016). The activity labeling and purification of PBA1 using Biotin-DEVD-FMK further confirmed that PBA1 possesses caspase-3-like activity (Pajerowska-Mukhtar and Dong, 2009; Salguero-Linares and Coll, 2019). PBA1 is involved in hypersensitive response (HR)-induced PCD in *Arabidopsis* leaves triggered by the pathogen *P. syringae*. A novel vacuolar membrane-plasma membrane fusion process participates in this mechanism and is regulated by PBA1. Downregulation of PBA1 blocks this membrane fusion and HR-induced PCD (Pajerowska-Mukhtar and Dong, 2009). The function of PBA1 in exhibiting caspase-3-like activity has been further validated in *Arabidopsis* (Ni et al., 2022). During xylem formation in poplar, caspase-3-like activity is closely associated with cell death in the xylem. Researchers purified caspase-3-like active proteins from the xylem and found that the 20S proteasome contributes to this activity (Han et al., 2012). The poplar 20S proteasome consists of seven α and seven β subunits. Although the specific subunit responsible for caspase-3-like activity was not identified in this study, consistent with previous findings, the caspase-3-like activity of the poplar 20S proteasome was significantly inhibited by β-lactone and aPnLd. Thus, these results indirectly support the role of PBA1 in caspase-3-like activity. In a study, downregulation of PBA1 expression was found to exacerbate endoplasmic reticulum stress-induced PCD, indicating that PBA1 acts as a negative regulator in this process (Cai et al., 2018).

## 9 The function of cathepsin B in plant PCD

### 9.1 Cathepsin B: A plant caspase-3-like protein

In 2016, a key breakthrough identified cathepsin B as a protease with caspase-3-like activity in plants, discovered through pull-down assays with Biotin-DEVD-FMK during UV-B-induced PCD (Ge et al., 2016). Cathepsin B belongs to the papain-like cysteine protease (PLCP) family (C1A subfamily) (Díaz-Mendoza et al., 2014; Richau et al., 2012). While animals have numerous “cathepsin” proteases (e.g., B, L, S), plants also possess these classes, though they are often named for their function (e.g., the cathepsin L-like protease SAG12, named for its role in senescence) (Díaz-Mendoza et al., 2014). *Arabidopsis thaliana* contains 33 C1A subfamily PLCPs (Díaz-Mendoza et al., 2014).

### 9.2 Synthesis and activation

Like other cysteine proteases, cathepsin B is synthesized as an inactive preproenzyme. This precursor contains an N-terminal signal peptide, prodomains, and a central catalytic domain. It is processed through the endoplasmic reticulum and Golgi apparatus, where the signal peptide is cleaved and glycosylation occurs, forming the inactive procathepsin B. Transport to the acidic environment of the vacuole triggers autoactivation, cleaving the prodomains to yield the mature, active enzyme (Richau et al., 2012; Mort and Buttle, 1997). While VPE is known to activate some PLCPs (Vorster et al., 2019), its role in cathepsin B activation remains unclear. The catalytic activity of mature cathepsin B depends on Cys and His residues (Ge et al., 2016; Porodko et al., 2018) and can exhibit endopeptidase, exopeptidase, and carboxypeptidase activities (Ge et al., 2016; Porodko et al., 2018; Tsuji et al., 2008; Niemer et al., 2016). In *Arabidopsis*, three genes (*CathB1, CathB2, CathB3*) encode cathepsin B, though *CathB1* produces inactive splice variants, suggesting *CathB2* and *CathB3* are the primary sources of enzymatic activity (Porodko et al., 2018; McLellan et al., 2009).

### 9.3 The role of cathepsin B in developmental PCD

Cathepsin B is an established regulator of PCD during various developmental processes:

- **Seed germination:** During *Arabidopsis* seed germination, *CathB3* is specifically highly induced (McLellan et al., 2009). *CathB3* T-DNA insertion mutants exhibit reduced cathepsin B activity and slower germination rates. The transcription factor G-Box Binding Factor 1 (GBF1) was identified as a negative regulator of *CathB3* expression (Iglesias-Fernández et al., 2014).
- **Leaf senescence:** *CathB3* is a marker for leaf senescence, and all three *CathB* genes are upregulated during dark-induced senescence (James et al., 2025). Triple *CathB* mutants show a delayed senescence phenotype, retaining higher chlorophyll levels and lower expression of the senescence gene *SAG12* than wild-type plants, confirming cathepsin B as a positive regulator of senescence (McLellan et al., 2009; Chou et al., 2018).

### 9.4 The role of cathepsin B in stress-induced PCD

Cathepsin B also modulates PCD triggered by biotic and abiotic stresses:

- **Biotic stress:** In the non-host hypersensitive response (HR) in tobacco, cathepsin B transcription and activity are induced by *Erwinia amylovora* (Eam). Both pharmacological inhibition and VIGS-mediated silencing of cathepsin B significantly suppressed Eam-induced and *Pseudomonas syringae*-induced PCD (Gilroy et al., 2007; Coppola et al., 2024). In *Arabidopsis*, *CathB2* and *CathB3* are upregulated by *Pst* DC3000, with triple *CathB* mutants showing reduced HR cell death, indicating functional redundancy among isoforms in pathogen defense (McLellan et al., 2009).
- **Abiotic stress:** The triple mutant *cathb#62* exhibits reduced PCD induced by UV-C and the oxidative stressor methyl viologen (MV) (Ge et al., 2016). During ER stress, cathepsin B and PBA1 both contribute to caspase-3-like activity but have antagonistic roles; loss of cathepsin B reduces cell death, while downregulation of PBA1 exacerbates it, highlighting the context-dependent functions of these proteases (Cai et al., 2018).
- **Low-temperature stress:** During barley microspore embryogenesis, which is induced by low temperatures and involves PCD, caspase-3 inhibitors reduce cell death. Concurrently, the activities and gene expression of cathepsin B, L/F, and H are elevated, suggesting a role for cathepsin B in cold-induced PCD (Bárány et al., 2018).

### 9.5 Substrates and mechanisms

Identifying cathepsin B substrates is critical for understanding its mechanism. In animals, cathepsin B promotes metastasis by degrading extracellular matrix proteins and facilitates autophagy by breaking down autophagosomal contents (Aggarwal and Sloane, 2014; Mijanović et al., 2019; Man and Kanneganti, 2016; Araujo et al., 2018; Wang et al., 2023). Recent plant studies have revealed that cathepsin B interacts with and degrades the large subunit of Rubisco (RbcL) (Yang et al., 2024), highlighting its direct role in protein degradation during plant PCD.

## 10 Application potential of caspase-like proteins in winter rapeseed genetic breeding

Winter rapeseed (*Brassica napus* L.) is a globally significant oilseed crop that plays a critical role in vegetable oil security while also serving as a valuable source of biofuel and animal feed. However, its productivity is persistently constrained by a spectrum of biotic and abiotic stresses, including fungal diseases such as *Sclerotinia sclerotiorum* and adverse environmental conditions including drought and cold (Yuan et al., 2026). Programmed cell death (PCD) is a fundamental, genetically regulated process essential for plant growth, development, and stress adaptation. Unlike animal apoptosis, which is primarily executed by a family of cysteine proteases known as caspases, plants have evolved a distinct but functionally analogous system of proteases—termed “caspase-like proteins” to orchestrate PCD (Iakimova et al., 2024). By manipulating these central regulators of cellular fate, it is possible to develop novel strategies for enhancing stress resistance, improving yield-related traits, and optimizing biotechnological breeding techniques.

### 10.1 Enhancing abiotic stress resistance

Manipulating the activity of caspase-like proteins offers a promising avenue for enhancing the tolerance of winter rapeseed to diverse abiotic stresses. Since many environmental adversities including drought, extreme temperatures, and high salinity either trigger PCD or are mediated by PCD pathways, fine-tuning the activity of these proteases can help plants maintain cellular homeostasis and survival under stress conditions.

Research in other plant species has firmly established a link between caspase-like proteins and abiotic stress responses. For instance, specific metacaspases in *Arabidopsis thaliana* have been implicated in drought response (Pitsili et al., 2023), while vacuolar processing enzymes (VPEs) are known to be induced by drought and contribute to stress responses in soybean nodules (Vorster et al., 2019). In rice, inhibition of the OsVPE3 gene enhances salt tolerance by reducing PCD-associated vacuolar rupture (Lu et al., 2016). These findings strongly suggest that orthologous genes in *Brassica napus* represent untapped genetic resources for improving stress resilience.

A prime target for such genetic engineering is the BnMCA gene family. A study on stress-induced microspore embryogenesis in *Brassica napus* demonstrated that transcriptional upregulation of several BnMCA genes including BnMCA-Ia, BnMCA-IIa, and BnMCA-IIi and a concurrent increase in MCA proteolytic activity were associated with cell death, thereby reducing the efficiency of haploid plant production (Berenguer et al., 2021). Pharmacological inhibition of MCA activity suppressed this cell death and increased the number of proembryos, indicating that MCAs function as pro-death factors in this context (Berenguer et al., 2021). This discovery provides direct proof-of-concept for manipulating MCA activity in rapeseed.

For field-grown winter rapeseed, which must overwinter in cold climates, strategies could be developed to modulate MCA activity specifically in sensitive tissues such as the root crown or leaves. By either suppressing the expression of specific BnMCA isoforms or engineering their proteolytic activity to be less sensitive to stress signals, it may be possible to reduce the extent of cold-induced PCD, thereby improving winter survival and spring regrowth vigor. Similarly, in drought-prone regions, targeted suppression of specific BnMCAs could prevent premature PCD of root cells or leaf mesophyll, thereby maintaining root function and photosynthetic capacity under water-limiting conditions (Ke et al., 2020; Shi et al., 2025; Yuan et al., 2026).

### 10.2 Improving biotic stress resistance and yield-related traits

The precise regulation of PCD is paramount for plant immunity, and caspase-like proteins occupy a central position in this process. Engineering disease resistance in winter rapeseed by manipulating these proteins holds significant potential, particularly against pathogens for which traditional resistance genes are limited.

The broad-spectrum necrotrophic fungus *Sclerotinia sclerotiorum* is a devastating pathogen of rapeseed, and current control measures remain insufficient. Recent research has identified a lesion mimic mutant, lmm1, in *B. napus* that exhibits enhanced resistance to *S. sclerotiorum*. This mutant displays spontaneous cell death lesions and shows constitutive upregulation of defense responses, including systemic acquired resistance (SAR) and elevated salicylic acid levels. This discovery directly links a controlled PCD phenotype to enhanced disease resistance and provides a valuable gene, LMM1, for marker-assisted breeding or transgenic approaches to introgress this resistance trait into elite rapeseed varieties (Yu et al., 2023).

Furthermore, the connection between leaf senescence a form of PCD and yield is well established. The timely breakdown of chlorophyll and other macromolecules in senescing leaves facilitates nutrient remobilization to developing seeds. In rapeseed, premature leaf senescence induced by stress can significantly reduce yield, whereas appropriately delayed senescence can prolong the photosynthetic period and increase biomass accumulation (Cui et al., 2023; Liu et al., 2023; Yan et al., 2021).

A functional study in rapeseed identified the BnaNAM transcription factor as a positive regulator of leaf senescence. Notably, overexpression of BnaNAM induced the expression of several senescence-associated genes, including proteases such as βVPE and γVPE, as well as the senescence-associated cysteine protease SAG12 (Wang et al., 2022). This finding provides a molecular link between a key transcription factor and the executioner proteases of PCD during senescence. By contrast, another study successfully constructed RNA interference (RNAi) vectors targeting a rapeseed cysteine protease gene, Bncp5, and generated transgenic plants with suppressed expression (Zhang et al., 2010). Although the specific function of Bncp5 in PCD was not detailed, this work validates RNAi as a tool for studying and manipulating protease function in rapeseed.

Therefore, a strategic approach to improving yield could involve fine-tuning the expression of key senescence-associated proteases such as VPEs. For example, promoters could be engineered to drive expression of BnVPE RNAi constructs in a senescence-enhanced but stress-induced manner, potentially generating varieties with an extended grain-filling period without negative pleiotropic effects on development.

### 10.3 Optimizing biotechnological breeding platforms

In addition to improving agronomic traits in the field, manipulation of caspase-like proteins offers powerful applications for accelerating breeding cycles through biotechnological methods. One such method is *in vitro* microspore embryogenesis, a technique used to produce doubled-haploid (DH) plants in a single generation, thereby significantly shortening the breeding timeline. However, a major bottleneck in this process is the spontaneous cell death of a large proportion of microspores following stress treatment, which drastically reduces the efficiency of embryo formation.

A landmark study in *Brassica napus* demonstrated that this cell death is mediated by both metacaspases and autophagy. Inhibition of either MCA activity or autophagy alone was sufficient to suppress cell death and increase the number of proembryos, while combined inhibition produced an even greater effect. This insight is directly translatable to applied breeding programs. Breeders can incorporate specific chemical inhibitors of MCA activity (e.g., Ac-YVAD-CMK) into the culture medium during the critical stress-induction phase of microspore embryogenesis. By transiently and safely blocking the central executioners of PCD, it should be possible to rescue a greater number of microspores, leading to higher yields of regenerable embryos and, consequently, more doubled-haploid lines for field evaluation.

This application has the potential to transform rapeseed breeding by making DH line production more efficient, cost-effective, and high-throughput. Furthermore, the genes identified in this study (e.g., *BnMCA-Ia*, *BnMCA-IIa*) serve as ideal molecular markers for selecting genotypes that are naturally less predisposed to PCD during *in vitro* culture, enabling rapid development of parental lines with superior response to biotechnological breeding (Berenguer et al., 2021; Pérez-Pérez et al., 2025).

## 11 Future perspectives and conclusion

While the potential of caspase-like proteins as breeding targets is considerable, several challenges must be addressed to translate this knowledge into successful commercial varieties.

### 11.1 Achieving spatiotemporal precision

A primary challenge is achieving precise spatiotemporal control over gene expression. Because PCD is essential for normal development, constitutive overexpression or knockout of these proteases could have severe negative consequences. The use of tissue-specific or inducible promoters—such as those responsive to stress, senescence, or chemical signals—will be crucial for confining genetic modifications to the desired temporal window and plant organ. For example, expressing an RNAi construct targeting a negative regulator of PCD specifically in guard cells could enhance drought tolerance without affecting growth in other tissues (Pitsili et al., 2023).

### 11.2 Addressing functional redundancy and network complexity

Another significant challenge lies in understanding the complex regulatory networks and functional redundancy among these proteases. These proteins do not act in isolation but are integrated into complex signaling networks involving Ca^2+^, reactive oxygen species (ROS), and post-translational modifications (Yamada et al., 2020; Li et al., 2012). Furthermore, gene families in *B. napus* are large and may exhibit substantial functional redundancy, with multiple VPE and MCA isoforms sharing overlapping roles. Future research should focus on generating higher-order mutants (e.g., double or triple knockouts) and employing systems biology approaches such as transcriptomics and proteomics to map the interaction networks of these proteases in rapeseed under diverse stress conditions (Tan et al., 2024; Fernández-Fernández et al., 2024).

### 11.3 Species-specific validation

Finally, the evolutionary divergence in substrate specificity among these proteases means that findings from model plants such as *Arabidopsis* must be validated directly in rapeseed (Savvateeva et al., 2015; Vercammen et al., 2004). The rapeseed genome is complex due to its recent allopolyploid origin, necessitating specialized bioinformatics tools and experimental approaches for accurate gene functional analysis.

### 11.4 Conclusion

In conclusion, the manipulation of caspase-like proteins represents a highly promising and multifaceted strategy for the genetic improvement of winter rapeseed. The accumulated evidence clearly indicates that these proteases are not merely executioners of cell death but serve as central hubs within the complex regulatory networks governing plant stress responses, development, and senescence. By strategically targeting metacaspases, VPEs, and other caspase-like proteins, breeders can potentially develop rapeseed varieties with enhanced resilience to both abiotic and biotic stresses, improved yield potential through optimized nutrient remobilization, and more efficient biotechnological breeding pipelines. Although challenges related to gene regulation, functional redundancy, and species-specific validation remain, the convergence of advanced molecular genetics, genomics, and precise genome-editing technologies provides an unprecedented opportunity to harness the power of PCD regulation for the benefit of global agriculture.
